# Design and Evaluation of pH-Sensitive Nanoformulation of Bergenin Isolated from *Bergenia ciliata*

**DOI:** 10.3390/polym14091639

**Published:** 2022-04-19

**Authors:** Kashaf Bashir, Muhammad Farhan Ali Khan, Aiyeshah Alhodaib, Naveed Ahmed, Iffat Naz, Bushra Mirza, Muhammad Khalid Tipu, Humaira Fatima

**Affiliations:** 1 Department of Pharmacy, Quaid-i-Azam University, Islamabad 45320, Pakistan; kashafbashir1@gmail.com (K.B.); farhanali6879@gmail.com (M.F.A.K.); natanoli@qau.edu.pk (N.A.); mktipu@qau.edu.pk (M.K.T.); 2 Department of Physics, College of Science, Qassim University, Buraydah 51452, Saudi Arabia; 3 Science Unit, Department of Biology, Deanship of Educational Services, Qassim University, Buraidah 51452, Saudi Arabia; i.majid@qu.edu.sa; 4 Department of Microbiology, Faculty of Biological Sciences, Quaid-i-Azam University, Islamabad 45320, Pakistan; 5 Department of Biochemistry, Faculty of Biological Sciences, Quaid-i-Azam University, Islamabad 45320, Pakistan; bushramirza@qau.edu.pk

**Keywords:** nanoparticles, stimuli-responsive polymer, Eudragit^®^, HPLC

## Abstract

The aim of the current study is extraction and isolation of bergenin from *Bergenia ciliata* and fabrication of pH-sensitive Eudragit^®^ L100 (EL100) polymeric nanoparticles (NP) to tackle limitations of solubility. Bergenin-loaded EL100 nanoparticles (BN-NP) were fabricated via nanoprecipitation and an experimental design was conducted for optimization. A reverse phase-high performance liquid chromatography (RP-HPLC) method was developed for the quantitation of bergenin. The optimized nanoformulation was characterized by its particle size, morphology, loading capacity, entrapment efficiency, drug–excipient interaction and crystallinity. An in vitro assay was executed to gauge the release potential of pH-sensitive nanoformulation. The mean particle size, zeta potential and polydispersity index (PDI) of the optimized nanoparticles were observed to be 86.17 ± 2.1 nm, −32.33 ± 5.53 mV and 0.30 ± 0.03, respectively. The morphological analysis confirmed the spherical nature of the nanoparticles. Drug loading capacity and entrapment efficiency were calculated to be 16 ± 0.34% and 84 ± 1.3%, respectively. Fourier transform infrared spectroscopy (FTIR) studies unfolded that no interaction was present between the drug and the excipients in the nanoformulation. Crystallography studies revealed that the crystalline nature of bergenin was changed to amorphous and the nanoformulation was stable for up to 3 months at 40 °C. The present study confirms that bergenin isolation can be scaled up from abundantly growing *B. ciliata*. Moreover, it could also be delivered by entrapment in stimuli-responsive polymer, preventing the loss of drug in healthy tissues.

## 1. Introduction

Since primitive times, humans have made use of natural products, such as animals, plants, microorganisms, and marine creatures in medicine to improve and treat ailments [1]. In the subsequent decade of the 19th century, several bioactive natural products were isolated from natural origins [2]. The exploitation of natural products in the development of new drugs, particularly in the quest for novel chemical structures, unfolded with remarkable success [3]. Specifically, drug discovery from medicinal plants can offer clues for therapeutically effective compounds in sapiens. Hence, there is unconstrained potential for forthcoming drug discoveries from plants which offers valuable information regarding novel chemical structures and their activities [4].

*Bergenia* genus belongs to the family *Saxifragaceae*: a small group of hardy perennials which grow as wild plants [5]. *Bergenia* comprises six species but the two most commonly available species include *B. stracheyi* and *B. ciliate* [6]. Due to the occurrence of countless therapeutically interesting primary and secondary metabolites, these plants have substantial biological properties and ethnopharmacological value [7]. *B. ciliata* (Haw) Sternb usually grows in moist and shady places, closely oppressed to rocks. Vernacular names of *B. ciliata* are Stone breaker, Batpia, Zahkm-e-hayat and Kachalu [8].

For a long time, *B. ciliata* has been acknowledged for its therapeutic potential and has been significantly used for a broad range of ailments in traditional systems [9]. Rhizome of *B. ciliata* has been used for curing pulmonary infections, piles, leucorrhea and for resolving kidney and bladder stones [10]. It has been used as a poultice for treating cough, fever, boils, menorrhagia, and severe uterine hemorrhage. Alcoholic extracts of the plant exhibit significant anti-inflammatory, analgesic, diuretic properties and anti-urolithiatic activity [11]. Methanol extract of rhizome exhibited considerable inhibition of cough reflex and displayed a broad spectrum of antibacterial activity. The parts of *B. ciliata* used for treatment of different ailments are the rhizome, stem, roots, latex, leaves, flower and whole plant [12].

Even though bergenin had been extensively utilized, its poor oral bioavailability is still a hindrance to its further application. Bergenin being a class IV compound, has neither enough solubility nor permeability for complete absorption [13]. Many efforts have been made for the improvement of its bioavailability, such as structure modification, prodrugs, and new dosage forms: β-cyclodextrin inclusion complexes and phospholipid complexes [14,15]. However, the enhancement of dissolution by the reduction in particle size of bergenin using nanosuspensions has been reported [16,17]. The nanoprecipitation method is used to encapsulate a large number of drugs [18]. It is easy to scale up and reproducible, which makes it one of the most frequently utilized approaches for nanoparticle preparation. It involves the preparation of organic and aqueous phases followed by the addition of the solvent phase into the continuously stirred non-solvent phase. The polymers used may be natural, synthetic, or semi-synthetic. Surfactants might be added into the formulation to avoid aggregation [19].

Several biodegradable and non-biodegradable pH-sensitive polymers are used for the encapsulation of drugs through nanoprecipitation [20]. Stabilizers introduce repulsive interactions by adsorbing on the surface of nanoparticles. Recently, stimuli-responsive nanocarriers, for instance, temperature and pH-sensitive nanoparticles, have gained attention due to their tremendous promises in diagnostic as well as therapeutic application [21]. Several pH-sensitive polymers, varying in their dissolution, and pH, have been used for the preparation of pH-responsive nanoparticles for numerous applications [22]. These polymers include synthetic methyl acrylate-methacrylic acid copolymers and cellulose-based semi-synthetic polymers such as Eudragit^®^ L100–55, L100, S100, cellulose acetate phthalate (CAP) and hydroxypropyl methylcellulose phthalate (HPMCP). In this study, Eudragit^®^ L100 (EL 100) was selected for nanoparticle preparation which may behave as pH-responsive due to the polymer [23].

Keeping in view the high cost, poor solubility and low permeability profile of bergenin as aforementioned, cost-effective isolation scale-up, exploiting indigenous resources, as well as nanotechnology-based improvement in pharmacokinetic parameters is direly needed. The aim of the current study is extraction and isolation of bergenin from *B. ciliata* and to design pH-sensitive Eudragit^®^ L100 nanoparticles. Furthermore, other objectives are characterization and identification of bergenin through spectroscopic analyses; preparation and optimization of potentially pH-sensitive nanoparticles of bergenin; investigation of the effect of independent variables (polymer concentration, surfactant concentration, and organic phase injection rate) on particle size and encapsulation efficiency and finally characterization of the nanoparticles for morphology, drug–excipient interaction, crystallinity, and stability.

## 2. Materials and Methods

### 2.1. Materials

Polyvinyl Alcohol (PVA) MW 146,000 (Sigma Aldrich, Milwaukee, Germany), Eudragit^®^ L100 (Evonik Chemicals, Essen, Germany), deionized water, methanol, *n*-butanol (Merck, Darmstadt, Germany) and ethyl acetate (Analytical grade, Lab-scan, Ireland) and methanol (HPLC grade, duksan chemicals, Ansan, Korea), drying oven (Contherm scientific Ltd., Lower Hutt, New Zealand), multi hotplate stirrer (WiseStir, Daihan Scientific Co., Ltd., Daegu, Korea), water distillation apparatus (IRMECO GmbH IM50, Aachen, Germany), centrifuge machine (Hermle, GmbH Z326k, Aachen, Germany), pH meter (pH 700, Eutech instruments, Loughborough, UK), refrigerator (Panasonic, MPR-161D H, Tokyo, Japan), ultrasonicator (Bransonic, 2210E-MT, Frankfurt, Germany), melting point apparatus, biomedical freezer (Panasonic, MDF-137, Tokyo, Japan), FTIR spectrometer (Perkin-Elmer, spectrum 65, Waltham, MA, USA), Scanning Electron Microscope (MIRA3, TESCAN, Brno, Czech Republic), Zeta sizer (Malvern ZS-1500, Malvern, UK), RP-HPLC (Perkin Elmer, Waltham, MA, USA), lyophilizer (Lyolab 3000, Copenhagen, Denmark), ultraviolet–visible spectrophotometer (Agilent Technologies, Palo Alto, CA, USA).

### 2.2. Methods

#### 2.2.1. Isolation and Extraction of Bergenin

*Bergenia ciliata* rhizomes were collected from Nathia Gali, Khyber Pakhtunkhwa, Pakistan. *B. ciliata* rhizomes were sorted to remove contamination and adulterations and shade dried in a well-ventilated room for 6 weeks until all the water content was removed. The dried rhizome was comminuted to a fine powder by using a commercial miller and stored in air-tight containers. Afterwards, the powdered rhizome was subjected to extraction. To prepare crude methanol extract, 2.5 kg powdered rhizome was transferred to large extraction jars. The ground rhizome was macerated with 7.5 L methanol for three days at room temperature, with occasional sonication in an ultrasonic bath for 30 min and thrice a day. The soaked material was strained and filtered using muslin cloth followed by filtration using Whatman no. 1. filter paper and the process was repeated in triplicate. The menstruum was combined and dried using a rotary evaporator at 45 °C to obtain crude methanol extract (CME) which was dried and stored in plastic jars until further use.

(1) $$\%\;\mathrm{Extract}\;\mathrm{recovery}=\frac{\mathrm{Total}\;\mathrm{weight}\;\mathrm{of}\;\mathrm{dried}\;\mathrm{extract}\;\mathrm{obtained}\;\mathrm{after}\;\mathrm{drying}}{\mathrm{Total}\;\mathrm{weight}\;\mathrm{of}\;\mathrm{dried}\;\mathrm{rhizome}\;\mathrm{taken}\;\mathrm{for}\;\mathrm{extraction}\;}\;\times \;100$$

The dried extract was weighed, and the percent recovery of CME was calculated by above-mentioned formula. Dried CME (400 g) was suspended in 600 mL distilled water in a large glass jar and shaken till homogenously dispersed. Ethyl acetate (800 mL) was added to it and gently mixed for 5–10 min. The mixture was let to stand for 30 min till the separation of the two layers after which the upper layer was removed by vacuum suction. Steps 2 and 3 were repeated 2 more times. Ethyl acetate extract was combined and concentrated through a rotary evaporator. The aqueous layer was extracted with *n*-butanol (500 mL). The *n*-butanol layer was separated by vacuum suction and concentrated through rotary evaporation. The final *n*-butanol fraction was evaporated in a high-efficiency vacuum evaporator. The remaining aqueous layer was also evaporated to half of the initial volume. Precipitates were allowed to settle. The precipitates were separated from both fractions and dissolved in a sufficient quantity of methanol. The methanol solution was filtered and slowly evaporated for bergenin crystallization. The yield of bergenin from *B. ciliata* rhizome was calculated as:

(2) $$\%\mathrm{yield}\;\mathrm{of}\;\mathrm{bergenin}=\frac{\mathrm{Total}\;\mathrm{weight}\;\mathrm{of}\;\mathrm{bergenin}\;\mathrm{isolated}}{\mathrm{Total}\;\mathrm{weigh}\;\mathrm{of}\;\mathrm{dried}\;\mathrm{rhizome}\;}\;\times \;100$$

#### 2.2.2. Identification of Bergenin

Bergenin (molecular weight; 328.27 g/mol) was characterized and identified based on its melting point and spectroscopic fingerprinting (UV–vis and IR spectroscopy). The obtained information was compared with the available data [24]. Bergenin (1 mg) was dissolved in 1 mL methanol and serially diluted in methanol to get a concentration of 25 µg/mL. Further, it was also analyzed on a UV–vis spectrophotometer at the wavelength range of 190–350 nm. Bergenin was engrained with KBr disc and scanned at an IR range of 400–4000 cm^–1^.

#### 2.2.3. Preparation of Bergenin-Loaded Nanoparticles

The nanoprecipitation technique was utilized for the preparation of nanoparticles. For aqueous phase preparation (10 mL), the surfactant PVA was taken in a beaker and dissolved in water through continuous stirring. Next, E L100 and bergenin (1 mg/mL) were separately dissolved in methanol and both solutions were combined to make the organic phase. The organic phase (2 mL) was then added dropwise into the continuously stirred aqueous phase, followed by the removal of the organic phase through rotary evaporation, resulting in the precipitation of nanoparticles in the aqueous phase. The resultant nanosuspensions were centrifuged at 13,500 rpm for 1 h to collect NP pellets which were washed with distilled water twice, lyophilized and stored for characterizations.

#### 2.2.4. Experimental Design for Optimization of Nanoparticles

Face-centered response surface methodology (RSM) was used in order to optimize the formulation using Design-Expert^®^ (Version 7.0.0, Minneapolis, MN, USA). Many designs are being available under RSM; however, Central Composite Design (CCD), being considered as most pertinent for 3 factors under response surface methodology (RSM), was utilized for the optimization of bergenin-loaded nanoparticles. The factors included in the study were polymer concentration, the amount of surfactant, and organic phase injection rate with low and high levels represented by −1 and +1, respectively, for which 15 runs were suggested by the software. Employed dependent variables for bergenin nanoparticles optimization, particle size and entrapment efficiency, were evaluated after the performance of each experiment. Constraints to be attained by the respective variables are displayed in Table 1.

Statistical analysis was performed by considering response surface analyses after selection of the most desirable mathematical model on the basis of statistical goodness of fit which includes F-value, predicted and adjusted R-square values and adequate precision. Data were analyzed through ANOVA (analyses of variance) after confirmation of model significance having a *p*-value < 0.05. Graphs were plotted using software and were helpful in observing the effect of independent variables on response variables. The 3-D response surface graphical view helps to analyze the effect of independent variables affecting the properties of dependent variables in a combined manner.

#### 2.2.5. Characterization of Prepared NPs

##### Mean Particle Size, Polydispersity Index and Zeta Potential

Analyses of hydrodynamic diameter, size distribution and zeta potential of all the 15 formulations were performed through a zeta sizer (Malvern ZS-1500) at 25 ± 2 °C for optimization of nanoparticles. For sample preparation, 10 µL of Bergenin-loaded EL100 nanoparticles (BN-NPs) were diluted up to 1 mL of double distilled water. Each analysis was performed in triplicate and expressed as mean ± SD.

##### Morphological Analysis

To observe the external morphology of the nanoparticles, samples were mounted on sample stub and sputtered with gold in sputter coater model SPI-MODULE (USA) for 90 s. at 30 mA. Images were taken by using SEM at the accelerating voltage of 20 keV.

##### Estimation of Percentage Yield, Entrapment Efficiency (EE) and Drug Loading (DL)

For the calculation of the total percentage yield of the nanoparticles, the initial quantities of the drug as well as the polymer were used. The yield was determined through the following formula [25];

(3) $$\%\;\mathrm{Yield}=\frac{\mathrm{Weight}\;\mathrm{of}\;\mathrm{dried}\;\mathrm{nanoparticles}\;}{\mathrm{Total}\;\mathrm{weigh}\;\mathrm{of}\;\mathrm{drug}\;+\;\mathrm{polymer}\;}\;\times \;100$$

The entrapment efficiency of all the formulations was calculated by using the indirect method. For this, the nanosuspensions were centrifuged at 13,500 rpm for 1 h at 25 °C in order to separate nanoparticles from the unentrapped drug and the free drug in the supernatant was quantified afterward using methanol. The encapsulation efficiency and drug loading were calculated through formulas [26];

(4) $$\%\;\mathrm{Entrapment}\;\mathrm{Efficiency}\;(\mathrm{EE})=\frac{\mathrm{Total}\;\mathrm{drug}\;\mathrm{added}-\mathrm{Free}\;\mathrm{drug}\;\mathrm{in}\;\mathrm{supernatent}}{\mathrm{Total}\;\mathrm{drug}\;\mathrm{added}}\;\times \;100$$

(5) $$\%\;\mathrm{Drug}\;\mathrm{Loading}\;(\mathrm{DL})=\frac{\mathrm{Weight}\;\mathrm{of}\;\mathrm{drug}-\mathrm{Free}\;\mathrm{drug}\;\mathrm{in}\;\mathrm{supernatent}}{\mathrm{Weight}\;\mathrm{of}\;\mathrm{nanoparticles}}\;\;\times \;100$$

##### Drug–Excipient Interaction

FTIR spectroscopy was performed using an FTIR spectrometer (Perkin-Elmer, spectrum 65, Waltham, MA, USA) to investigate any potential interaction between the drug and excipients. Bergenin, EL 100, the physical mixture (BN + EL 100) and the final formulation were analyzed through pellets preparation by mixing them separately with KBr in a ratio of 1:100 through the application of lb. pressure. Pellets after being placed in the sample holder were scanned at a wavelength range of 4000–400 cm^–1^.

##### X-ray Diffraction

XRD analysis was performed to check the amorphous or crystalline nature of individual ingredients used in the preparation of nanoparticles, i.e., Bergenin, EL100, physical mixture (BN + EL 100) as well as nanoformulation. For this purpose, XRD was applied at room temperature (20 kV, 5 mA). After that absolute intensity was recorded in the range of 5–80 against theta-2.

##### In Vitro Drug Release Studies

In vitro release studies of NPs formulation and free drug dispersion were carried out in phosphate buffer at pH 7.4, 6.4 and 5.5, using a dialysis membrane (having no binding potential and molecular weight cutoff 14,000 Da). The formulation was placed in a dialysis sac to evaluate medication release over time. This medium was placed on a shaking water bath at 37 °C after dipping a dialysis sac in a beaker. All of the experiments were performed in triplicate. The time intervals for sampling were 0.5, 1, 2, 3, 4, 5, 6, 8, 10, 12, 24, 36, 48 and 72 h. At each time interval, 1 mL of sample was taken. To keep the sink conditions, phosphate buffer of the appropriate pH was used to replace the amount of sample taken. At a wavelength of 219 nm, a UV–visible spectrophotometer was used to quantify these samples. Using MS Excel, a graph of percentage drug release vs. time was created to calculate drug release. The release profile of pH-sensitive nanoformulation was evaluated using first order, zero order, Higuchi, Hixon–Crowel, and Korsmeyer Peppas kinetic models [18].

##### Physical Stability Study

The physical stability study of the nanoformulation on storage was studied at 5 ± 3, 25 ± 2 and 40 ± 2 °C (75 ± 5% RH) for up to 3 months. The physical stability was then evaluated by taking particle size measurements, which were selected as suitable parameters.

## 3. Results

### 3.1. Extraction Efficiency and Bergenin Yield

A total of 417 g of crude methanol extract was obtained from 2.5 kg of dried powdered *B. ciliata* rhizome. Sonication aided maceration was used as an extraction technique and 16.68% extract recovery was calculated. CME was subjected to solvent–solvent extraction and 12.7 g of bergenin was recovered after crystallization. Recovery of bergenin was calculated to be 0.5% *w*/*w* of dry rhizome powder.

#### 3.1.1. Identification of Bergenin

**Figure 1:**
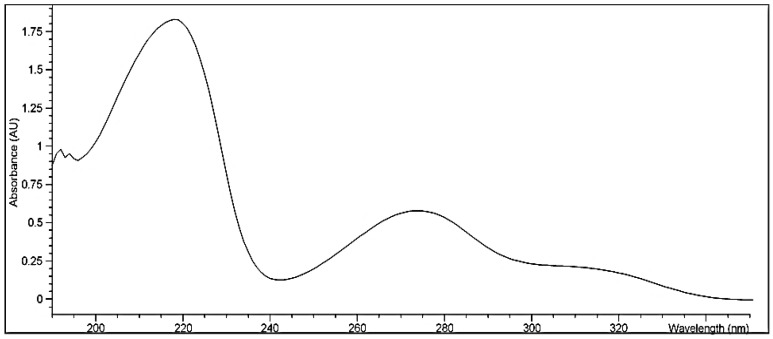
UV–vis spectrum of bergenin isolated from *B. ciliata*.

The melting point of the bergenin was observed to be 236 °C. The UV–visible spectrum of bergenin dissolved in methanol showed λ_max_ at 219 and 274 nm (Figure 1).

#### 3.1.2. FTIR Spectroscopy of Bergenin

**Figure 2:**
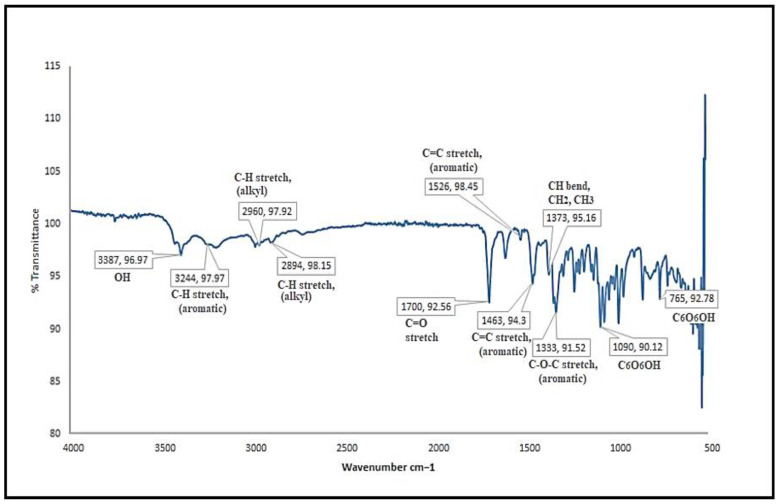
FTIR spectra of bergenin isolated from *B. ciliata*.

The FTIR (ν_max_ cm^−1^) spectra of isolated bergenin (Figure 2) shows characteristic 3387 (O−H); 3244 (C−H stretching, aromatic); 2960, 2894 (C−H stretching, alkyl); 1700 (C=O stretching); 1609, 1526, 1463 (C=C stretching, aromatic); 1373 (CH bend, CH_2_, CH_3_); 1333 (C−O−C stretching, ether); 1090, 765 (C_6_H_6_OH). The isolated crystalline compound was confirmed as bergenin when the melting point, UV and FTIR spectra were compared with the data reported for standard bergenin.

### 3.2. Preparation of Optimized Formulation Using Factorial Design

Utilizing Central Composite Design (CCD) under Design-Expert^®^ software version 7.0.0, 15 formulations with varying concentrations of polymer and surfactant were successfully formulated and screened as described earlier in the previous section. Coded values of A, B, and C were given by the software to EL 100, PVA, and organic phase injection rate, respectively. A quadratic equation was generated according to the coded values (A, B, C) for particle size (nm) and entrapment efficiency (%). Further 3-D response surface graphs helped to depict the responses in terms of predetermined factors. After careful examination, the effect of each independent variable on various responses was determined. The dependent responses: mean particle size (nm) and entrapment efficiency (%) of all 15 formulations were analyzed. Dependent responses for each individual trial with corresponding values of independent variables are depicted in Table 2.

#### 3.2.1. Response Effects of Independent Variables on Particle Size and Entrapment Efficiency

##### Effect of Independent Variables on Particle Size (Y1)

**Figure 3:**
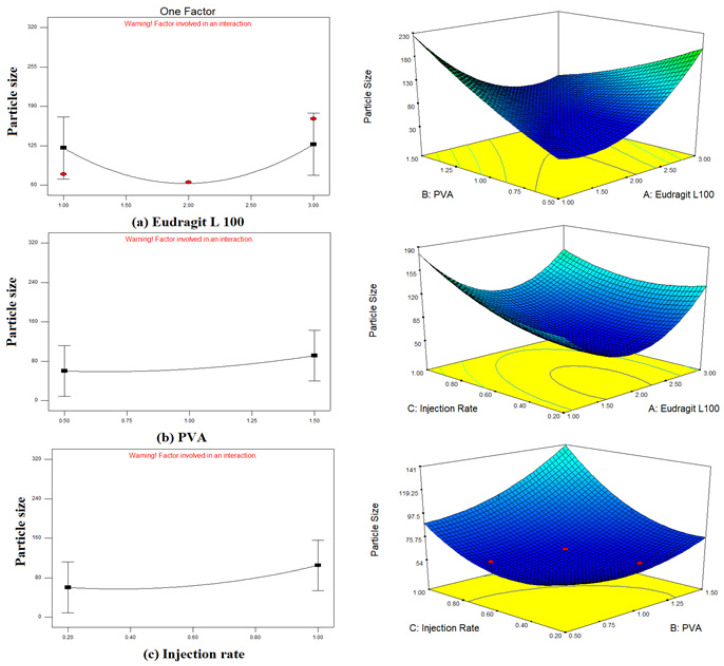
Graphical representation of the impact of independent variables on mean particle size of bergenin-loaded nanoparticles. (**a**) EL 100 (**b**) PVA (**c**) organic phase injection rate and 3-D response surface plot (**a**) PVA and EL 100; (**b**) organic phase injection rate and EL 100; (**c**) organic phase injection rate and PVA.

The particle size and EE of the 15 formulations are illustrated in Table 2. In order to obtain the relation between particle size and the independent variables, various mathematical models; mean, linear, quadratic, 2F1 and cubic models were analyzed for predicting the best fit model using the software. So, on the basis of the *p*-value of ˂0.05, the quadratic model was selected for analyses of particle size. The average particle size of formulations was within the nano-metric range from 61 nm to 319 nm. Three-dimensional surface plots clearly explain the impact of polymers concentration, surfactant concentration and organic phase injection rate on the mean particle size of nanoparticles as demonstrated in Figure 3.

##### Effect of Independent Variables on Entrapment Efficiency (Y2)

**Figure 4:**
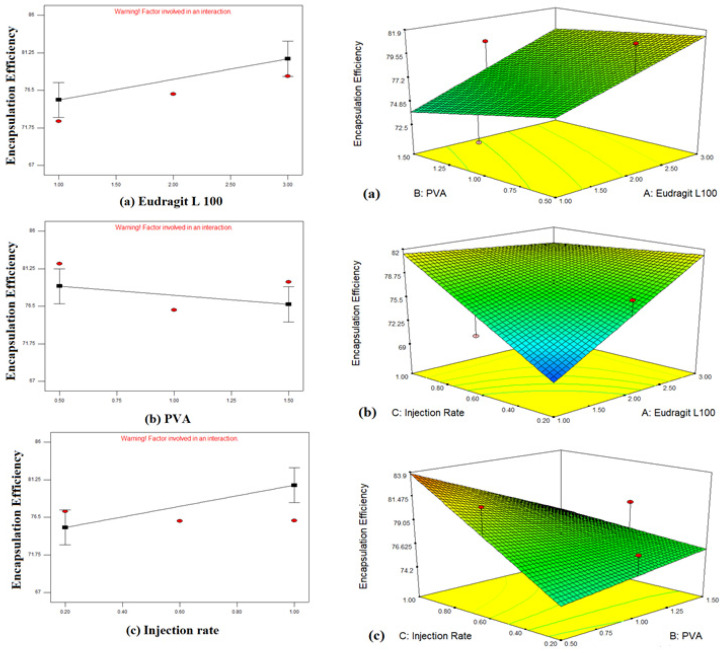
Graphical representation of the impact of independent variables on encapsulation efficiency of bergenin-loaded nanoparticles. (**a**) EL 100 (**b**) PVA (**c**) organic phase injection rate and 3-D response surface plot (**a**) PVA and EL 100 (**b**) organic phase injection rate and EL 100 (**c**) organic phase injection rate and PVA.

The entrapment efficiency of 15 formulations of bergenin nanoparticles ranges from 67.66 to 85.5%. The correlation between entrapment efficiency and independent variables was drawn after the selection of a suitable model based on a *p*-value of <0.05. The 2FI was selected having a *p*-value of 0.0322. Three-dimensional surface graphs explaining the impact of polymers, surfactants and injection rates on entrapment efficiency are depicted in Figure 4.

#### 3.2.2. Selection and Preparation of Optimized Nanoformulation

**Figure 5:**
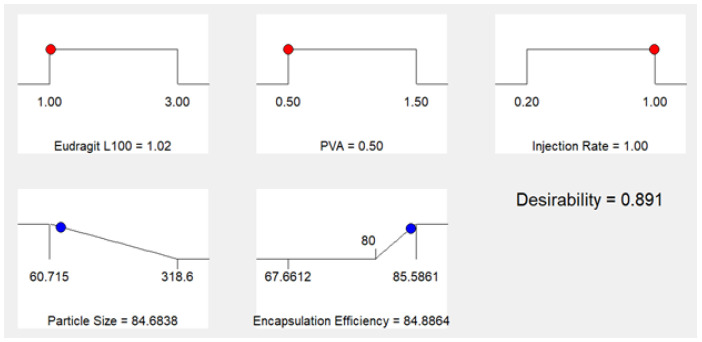
Values generated by design expert for development of optimized nanoformulation.

Optimized formulation was developed after the evaluation of 15 formulations in terms of their mean particle size and entrapment efficiency. Optimized formulation was developed using 50 mg or 1.02% (*w*/*v*) EL 100, 0.5% (*w*/*v*) PVA and an injection rate of 1 mL/min as suggested by the software having desirability of 0.891, i.e., having 89.1% chance of producing the required results, predicted by the software as shown in Figure 5.

Further analyses and studies were performed for the optimized formulation. Based on these results, optimized bergenin nanoformulation with a mean particle size of approximately 84.68 nm and an entrapment efficiency of 84.8% was chosen for future studies.

### 3.3. Characterization of Optimized Formulation

#### 3.3.1. Size Measurement and Zeta Potential

**Figure 6:**
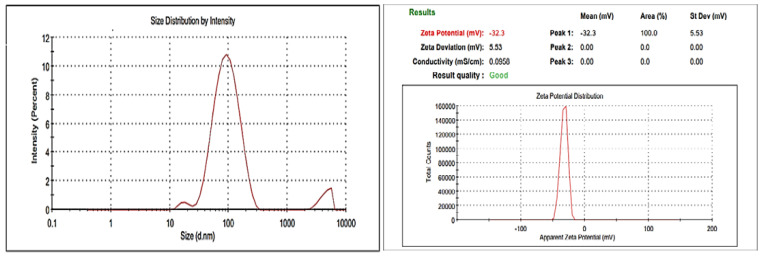
Particle size, PDI and Zeta potential measurement of optimized nanoformulation.

The smallest particle size of the optimized formulation is desirable with minimum PDI to keep the formulations stable. The particle size and PDI of optimized bergenin-loaded NPs are presented in Figure 6. Readings were taken in a triplicate manner and expressed as (*n* = 3, mean ± SD). The mean particle size for optimized nanoformulation was observed to be 86.17 ± 2.1 nm with a PDI of 0.30 ± 0.03. Zeta potential is a parameter that measures the surface charge of the formulation and is the measure of the stability of the nanodispersions. Absolute high potential values (±30 mV) are preferable in most cases in order to create a high energy barrier between the particles preventing their coalescence. The zeta potential value of the optimized nanoformulation was −32.33 ± 5.53 and is presented in Figure 6.

#### 3.3.2. Morphological Analyses

**Figure 7:**
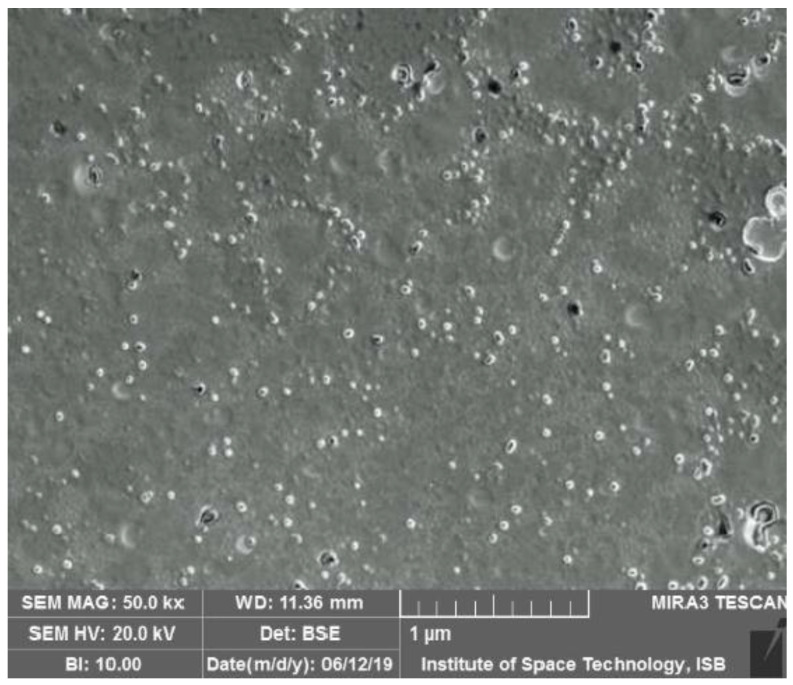
SEM image of optimized nanoformulation.

The morphology of precipitated nanoparticles particles is shown in Figure 7. The precipitated nanoparticles with the PVA as stabilizer were spherical in shape and uniformly distributed.

#### 3.3.3. Quantification of Bergenin through RP-HPLC

**Figure 8:**
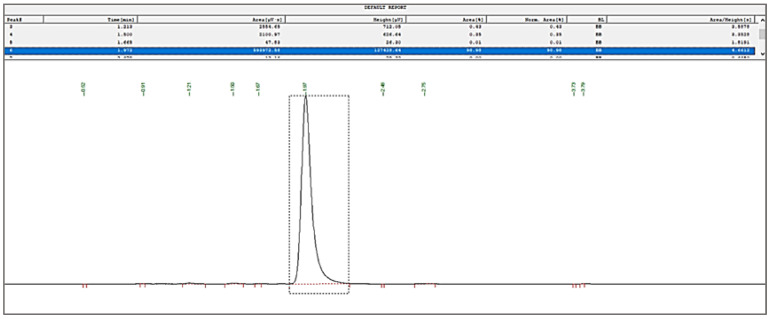
HPLC chromatogram of bergenin.

HPLC protocol was optimized for the quantification of bergenin from its nanoformulations in the present study. The HPLC chromatogram of bergenin at 6.25 (µg/mL) is shown in Figure 8.

#### 3.3.4. Fourier Transform Infrared Spectroscopy (FTIR)

**Figure 9:**
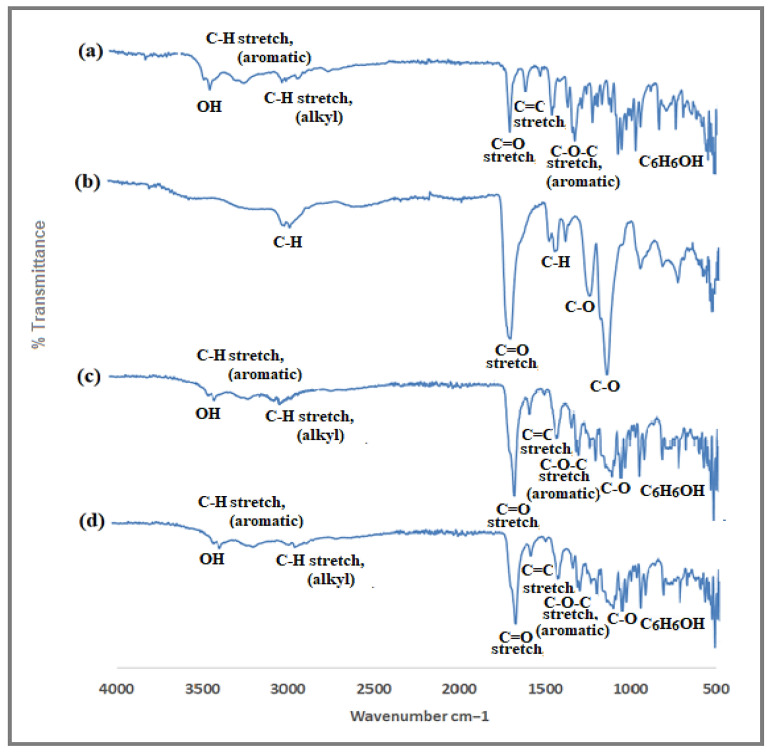
FTIR spectra of drug, polymer, physical mixture and nanoformulation. (**a**) Bergenin (**b**) EL 100 (**c**) lyophilized nanosuspensions (**d**) physical mixture of bergenin + E L100.

As presented in Figure 9, there are characteristic peaks of bergenin at 3387 cm^−1^ (O−H); 3244 cm^−1^ (C−H, aromatic); 2960, 2894 cm^−1^ (C−H, alkyl); 1700 cm^−1^ (C=O); 1609, 1526, 1463 cm^−1^ (C=C, aromatic); 1373 cm^−1^ (CH bend, CH_2_, CH_3_); 1333 cm^−1^ (C−O−C, aromatic); 1090, 765 cm^−1^ (C_6_H_6_OH) whereas EL100 spectrum exhibits peaks at 2900, 1450, 1390 cm^−1^ (CH), 1705 (C=O), 1150 and 1250 cm^−1^ (esterified carboxyl groups). New peaks or changes were not observed in the physical mixture. However, the characteristic absorption peaks of bergenin were visible in the spectrum of the final formulation, and the absence of new peaks indicated no interaction.

#### 3.3.5. X-ray Diffraction Studies (XRD)

**Figure 10:**
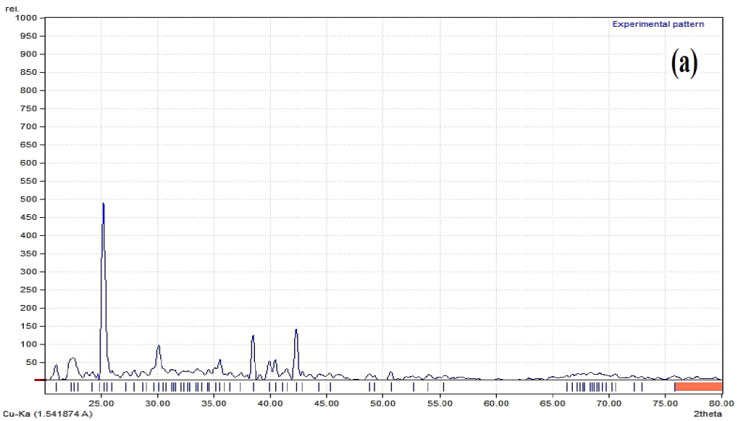

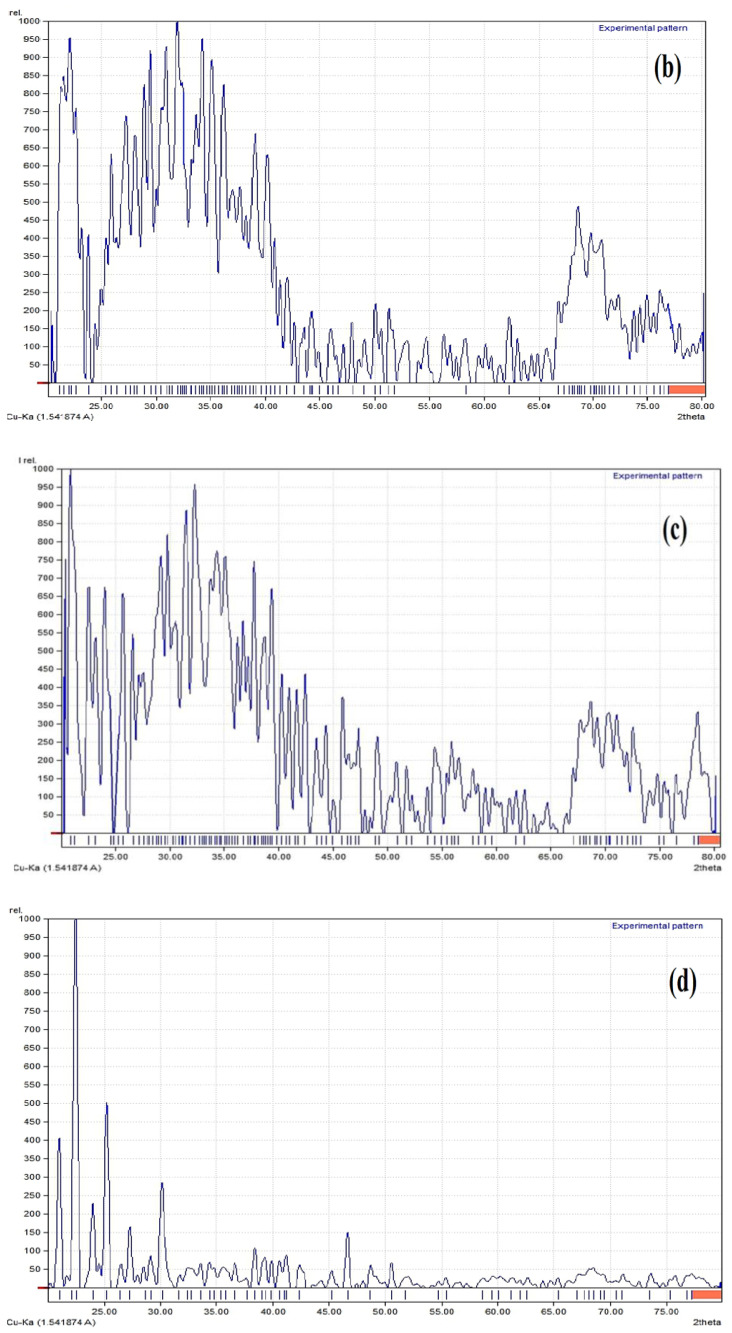
XRD spectra of drug, polymer, physical mixture and nanoformulation (**a**) bergenin; (**b**) EL 100; (**c**) lyophilized nanosuspension; (**d**) physical mixture of bergenin + E L100.

Pure bergenin has intense diffraction peaks, attributable to its crystalline nature. These peaks were also evident in the physical mixture. However, no peaks of the drug were detected in optimized nanoformulation which indicates that the crystalline form of the drug was changed to amorphous (Figure 10).

#### 3.3.6. In Vitro Release

**Figure 11:**
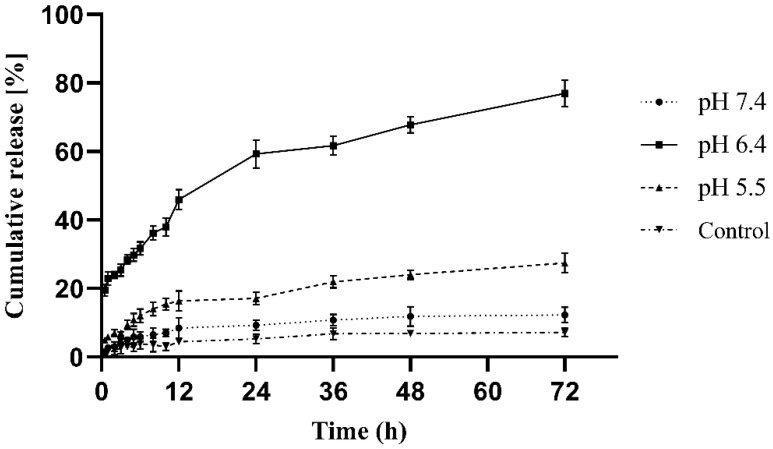
In vitro release profile of pH-sensitive nanoformulation at pH 7.4, 6.4, 5.5.

At pH 7.4, 6.4 and 5.5, a dialysis membrane technique was used to assess drug release from pH-sensitive nanoformulation. Free drug dispersion as a control in the release experiments exhibited negligible release. Figure 11 shows that nanoformulation released roughly 76 percent of all entrapped medication in 72 h at pH = 6.4, whereas drug release was 27 percent in 72 h at pH = 5.5. However, at pH = 7.4, drug release was negligible.

#### 3.3.7. Physical Stability Study

The physical stability of storage of the optimized nanoformulation was observed after 3 months. The formulation remained stable at 5 ± 3, 25 ± 2 and at 40 ± 2 °C/ 75 ± 5% RH for up to 3 months. However, a significant rise in particle size was observed in those formulations kept at 40 °C for three months. The surfactant PVA in a concentration of 0.5% appeared to be sufficient for providing proper steric coverage to the suspended nanoparticles. The particle size diameter data on storage are given in Table 3. No sedimentation was observed in the optimized formulations during the period of 3 months which means that the nanoformulation was stable.

## 4. Discussion

Plants have been extensively used in diverse human fields including their use as food and medicine [27]. The therapeutic potential of medicinal plants has been observed and acknowledged for a long time in order to develop satisfactory treatment measures for various diseases [28]. A number of molecules have been identified and isolated from plants possessing strong pharmacological potential [29]. The undertaken study was planned for extraction optimization of *B. ciliata* for efficient bergenin isolation, utilizing non-complex extraction and isolation techniques. Extraction and isolation of compounds of interest from plants depend on many factors including the type of plant material, nature of extraction medium and method applied. Successive solvent extraction method assisted by intermittent ultra-sonication is a preferred method for crude extracts and was performed in the current study [30]. Reduced extraction time, solvent usage, least sample degradation and improved extraction yield are the advantages that make this method superior to the conventional method of extraction.

Through an extensive literature survey, it was found that rhizome is the most frequently (43%) utilized part of *B. ciliata* [10]. Its various ethnomedicinal uses including the treatment of fractured bones, sores, wounds, fresh cuts, diarrhea, fever, pulmonary infections, vomiting, boils and cough [31]. Bergenin, commonly known as cuscutin, is a C-glucoside of 4-O-methyl gallic acid. It is abundantly found in the genera of Saxifragaceae, Myrsinaceae and Euphorbeaceae. It is a colorless crystalline polyphenol isolated from Bergenia species including *B. ciliata*, *B. ligulata*, *B. crassifolia*, *B. stracheyi*, etc. It is the major compound present up to 0.6% in the rhizome of *B. ciliata*. Furthermore, *B. ciliata* rhizome was reported to have the highest bergenin content among all the other parts [32]. So, it was chosen for the extraction optimization and isolation of bergenin. The methanol extract of *B. ciliata* was reported to have the maximum bergenin content and was chosen for further processing of bergenin isolation [33].

The percentage yield of isolated bergenin was 0.5% of the total dried *B. ciliata* rhizome. It was slightly variable with the reported yield of 0.6% from the same plant [32]. The difference observed in bergenin yield may be due to the variability of the region from which the plant was collected, and the use of new extraction and isolation techniques. Bergenin was isolated as colorless crystals. The melting point of 237 °C was consistent with the published melting point of bergenin at 236 °C [12]. UV–visible spectrum of bergenin showed λ_max_ at 219 and 274 in accordance with the published data. The IR spectra of isolated bergenin showed characteristic peaks having no interaction [14,34].

The solubility of bergenin in different solvents was checked by dissolving it in water, methanol, ethanol and DMSO. It was observed that bergenin was soluble in methanol and DMSO, however, had no solubility in water and ethanol in accordance with previous findings [35]. To overcome the low solubility of bergenin in water, its nanoformulation was designed. For this purpose, EL100-based pH-sensitive nanocarriers containing the model drug, i.e., bergenin were prepared. Stimuli-responsive polymers present remarkable benefits in drug delivery. Despite behaving passively, these polymers interact and respond to the environmental setting more precisely and intelligently. These pH-sensitive nanocarriers aim to deliver the model drug to the target site without being released on healthy tissues. The reason for the use of EL100 for targeted drug delivery includes its pH-dependent dissolution behavior, the polymer being insoluble at pH < 6 while soluble at pH > 6. The importance of pH-sensitive polymers is that drug release from NPs within a restrained environment is a major problem and pH-sensitive NPs have the potential to solve this problem [21].

The nanoprecipitation method was used for the preparation and optimization of bergenin-loaded Eudragit^®^ L 100 nanoparticles because both the drug and the polymer utilized in this study are hydrophobic in nature and this method is most suitable for such types of drugs [36]. Furthermore, this method is reproducible, requires a minimum number of excipients and gives the desired results for our nanoformulation. The yield of the optimized nanoformulation as calculated by the indirect method was found to be 63.04 ± 1.60%. The encapsulation efficiency and drug loading of the formulation were calculated to be 84.12 ± 1.30 and 15.23 ± 0.34%, respectively. Similar results have been previously reported using the same preparation technique [37].

Fifteen different trials were generated by the Design-Expert^®^ software version 7.0.0. It was observed that as the concentration of PVA was increased from 0.5 to 1.5% the particle size increased from 61 to 319 nm. Such an effect of PVA concentration on particle size has also been reported [38]. Budhian suggested the existence of two contending effects at higher polyvinyl alcohol concentration, i.e., a higher concentration of surfactant prompted increased solubility at the interface of two phases leading to particle size reduction and higher viscosity leads to inefficient mixing of two phases resulting in particle growth [39]. It was observed that the particle size decreased by increasing the polymer concentration from 0.5 to 1% *w*/*w* but started increasing when the polymer concentration was further increased. When the polymer concentration is increased it favors particle growth with respect to particle nucleation, hence increasing the overall particle size [40].

The encapsulation efficiency of these trials ranged from 67 to 85%, and it decreased with the increasing PVA concentration and increased with increasing the EL100 concentration and organic phase injection rate. The reason for the increase of the encapsulation by increasing polymer concentration can be assumed as, by increasing the polymer concentration, large-sized particles are produced leading to higher drug entrapment [19]. Whereas, increasing PVA concentration results in a decrease in encapsulation efficiency. The reason could be the addition of a larger concentration of surfactant to the aqueous phase produces cavitation causing leakage of drug from the continuous phase to the external phase hence lowering encapsulation efficiency [41]. The other reason can be higher PVA concentration repels the force applied to the system causing an increment in particle size.

Based on the preferred responses, design expert enables the researcher to design and chose an optimized formulation. In the present work, our purpose of using design expert was to prepare NPs with minimum particle size and maximum encapsulation efficiency [42]. On the basis of the desirability of maximum EE and minimum particle size, design expert proposed one formulation (1% *w*/*w* EL 100, 0.5% *w*/*w* PVA and 1 mL/min organic phase injection rate). Hence, the optimized formulation was prepared using these values for the variables. The average particle size of the optimized bergenin nanoformulation was 86.17 ± 2.1 nm with a PDI of 0.3 ± 0.03. Morphological analysis confirmed the particles to be spherical, uniformly distributed and no sign of agglomeration was observed. Similar results have been reported by using a polymer as a stabilizer.

FTIR analyses showed that there was no chemical interaction between the ingredients of NPs (as indicated by physical mixture analyses) as well as in the nanoparticles, as the absence of any functional group or new peak was not observed. Similar results by the use of EL100 and PVA as excipients have been reported [35]. The XRD patterns of the pure bergenin displayed the presence of numerous distinct peaks at 25°, 30°, 36°, 39° and 42°, which suggested that the drug was in crystalline form. The precipitated nanoparticles samples showed diminished peaks suggesting the conversion of crystalline bergenin into an amorphous form upon precipitation into nanoparticles which is in correlation with the previous findings [43].

The mechanism of drug release was determined using various release models such as zero order, first order, Hixon–Crowel and Korsmeyer Peppas. In vitro release studies of prepared NPs and free drug dispersion exhibited minute release at pH = 7.4, pH = 6.4 and pH = 5.5; however, NPs at pH = 6.4 had shown better release: significant difference in release pattern confirming that the drug has been successfully encapsulated in pH-sensitive polymer, i.e., EL^®^100. The in vitro release from the polymeric matrix at pH = 6.4 was found to be in a sustained manner. The reason behind this might be that as the pH of the release medium becomes above 6, carboxylic functional groups of the polymer become ionized bringing structural changes as well as swelling of the polymer matrix resulting in the release of drug from the network structure. R2 data show that the release follows the Korsmeyer Peppas model. The R2 coefficient was 0.991. The value of *n* in the Korsmeyer Peppas model is critical for investigating the drug transport mechanism. For the calculation of this value, only the first 60% of the release is taken into account. The fact that drug release from nanoparticles follows a pH-dependent release is explained by an R2 value near 1. This model explains the non-Fickian drug release from the pH-sensitive nanoformulation system [18].

Stability studies were conducted according to ICH guidelines and the effect of temperature and relative humidity on the stability of the nanoformulation was checked. At different temperature conditions of 5 ± 3°, 25 ± 2° and 40 ± 2 °C/ 75 ± 5% RH, the formulation remained stable at 5 ± 3, 25 ± 2 for up to three months. However, a significant rise in particle size was observed in those formulations kept at 40° C for three months but the increase in the particle size was from 86 to 98 nm showing no big change which can affect the properties of the system. The surfactant PVA in a concentration of 0.5% appeared to be sufficient for providing proper steric coverage to the suspended nanoparticles. The particle size diameter data on storage are given in Table 3. No sedimentation was observed in the optimized formulations during the period of 3 months which means that the nanoformulation was stable. [44].

## 5. Conclusions

Colorless bergenin crystals with a yield of 0.5% *w*/*w* have been successfully obtained from non-complex extraction and isolation techniques from the crude methanol extract. Furthermore, Bergenin-loaded Eudragit^®^ L100 has been successfully prepared and optimized using Design-Expert^®^ software version 7.0.0. The effect of independent variables (polymer concentration, surfactant concentration and organic phase injection rate) on particle size and encapsulation efficiency was also evaluated giving a particle size range of 61–319 nm and encapsulation efficiency range 68–86%. The optimized nanoformulation was selected based on its particle size (86.17 nm) and polydispersity index (0.30). It has been successfully characterized for particle size, zeta potential, morphology, drug–excipient interaction, solid-state form, in vitro release and stability. *B. ciliata* could be utilized as a commercial source of bergenin. Moreover, optimized nanoparticles might be loaded into hydrogel for topical application, keeping in view its anti-inflammatory and wound healing properties. Finally, in vivo studies will provide further insights into the bioavailability profile of bergenin-loaded nanoformulation.

## Figures and Tables

**Table 1:** Variables in CCD for bergenin nanoparticles and development.

Independent Variables	Levels	
	−1	+1
X1: EL100 *w*/*v* (%)	1.0	3.0
X2: PVA *w*/*v* (%)	0.5	1.5
X3: Inj. rate (mL/min)	0.2	1
**Dependent Variables**	**Goal**	
Y1: Mean particle size (nm)	Minimize	
Y2: Entrapment efficiency (%)	Maximize	

**Table 2:** Particle size and entrapment efficiency of 15 runs designed by the software.

Runs	Independent Variables			Dependent Variables	
	X1	X2	X3	Y1	Y2
	EL100 *w*/*v* (%)	PVA *w*/*v* (%)	Inj. Rate (mL/min)	Particle Size (nm) (Mean ± SD)	Encapsulation (%) (Mean ± SD)
1	2	1	0.6	65 ± 2.30	76 ± 0.13
2	3	1.5	0.2	85 ± 3.20	83 ± 0.90
3	1	1.5	0.2	191 ± 2.10	68 ± 1.20
4	1	1	0.6	78 ± 1.90	73 ± 0.70
5	3	0.5	0.2	188 ± 2.20	78 ± 0.50
6	1	0.5	0.2	61 ± 2.50	70 ± 1.10
7	2	1	0.2	68 ± 3.30	77 ± 0.90
8	2	1	1	94 ± 2.70	76 ± 1.20
9	2	1.5	0.6	79 ± 4.00	80 ± 1.10
10	2	0.5	0.6	69 ± 2.80	82 ± 1.00
11	3	1.5	1	95 ± 3.40	76 ± 0.90
12	3	1	0.6	170 ± 2.10	78 ± 0.70
13	3	0.5	1	220 ± 1.60	86 ± 0.30
14	1	0.5	1	78 ± 2.30	84 ± 1.10
15	1	1.5	1	319 ± 3.10	81 ± 0.50

Values are presented as the mean (*n* = 3) ± SD.

**Table 3:** Physical stability evaluation of the bergenin loaded nanosuspension.

Formulation	Storage Temperature Conditions	Initial Particle Size (nm)	Particle Size After 1 Month (nm)	Particle Size After 2 Months (nm)	Particle Size After 3 Months (nm)
Optimized Formulation	5 ± 3 °C	86.17 ± 2.1	86.7 ± 2.2 ^a^	87.6 ± 2.5 ^a^	88.9 ± 2.8 ^a^
	25 ± 2 °C 65 ± 5% RH		86.5 ± 3.2 ^a^	88.1 ± 3.5 ^a^	90.4 ± 3.7 ^a^
	40 ± 2 °C/ 75 ± 5% RH		89.5 ± 3.0 ^a^	93.6 ± 4.2 ^a^	98.2 ± 4.6 ^b^

**Note:** Values are presented as the mean (*n* = 3) ± SD. All mean were analyzed by two-way ANOVA using LSD for multiple comparisons. The values having different superscripts in the same row are significantly different (*p* ≤ 0.05).

## Data Availability

Not applicable.
